# Analysis of mRNA processing at whole transcriptome level, transcriptomic profile and genome sequence refinement of *Trypanosoma cruzi*

**DOI:** 10.1038/s41598-019-53924-6

**Published:** 2019-11-22

**Authors:** Francisco Callejas-Hernández, Ángel Gutierrez-Nogues, Alberto Rastrojo, Núria Gironès, Manuel Fresno

**Affiliations:** 1 Centro de Biología Molecular Severo Ochoa, Consejo Superior de Investigaciones Científicas, Universidad Autónoma de Madrid, Cantoblanco, Madrid, Spain; 2 Instituto Sanitario de Investigación Princesa, Madrid, Spain

**Keywords:** Transcriptomics, Gene expression, Gene regulation

## Abstract

The genomic sequence of *Trypanosoma cruzi*, the protozoan causative of Chagas disease was published more than a decade ago. However, due to their complexity, its complete haploid predicted sequence and therefore its genetic repertoire remains unconfirmed. In this work, we have used RNAseq data to improve the previous genome assembly of Sylvio X10 strain and to define the complete transcriptome at trypomastigote stage (mammalian stage). A total of 22,977 transcripts were identified, of which more than half could be considered novel as they did not match previously annotated genes. Moreover, for the first time in *T. cruzi*, we are providing their relative abundance levels. We have identified that Sylvio X10 trypomastigotes exhibit a predominance of surface protein genes, specifically those encoding trans-sialidase and mucin-like proteins. On the other hand, detailed analysis of the pre-mRNA processing sites revealed some similarities but also some differences in the spliced leader and different polyadenylation addition sites compared to close related kinetoplastid parasites. Our results also confirm that transcription is bidirectional as occur in other kinetoplastids and the proportion of forward-sense and reverse-sense transcripts is almost equivalent, demonstrating that a strand-specificity does not exist.

## Introduction

*Trypanosoma cruzi* is a protozoan parasite causative of Chagas disease, bearing the name of its discoverer the Brazilian scientist Carlos Chagas. This parasite has a complex life cycle that includes an insect vector and a broad range of mammalian hosts, including domestic animals and sylvatic reservoirs^1,2^ and therefore, depends on several key points of cellular reprogramming through its entire life cycle to adapt to the different biological enviroments^3^. The insect vector is infected by non-replicative trypomastigotes present in the blood of mammalian reservoirs and differentiate into replicative epimastigotes in the midgut of the vector. Next are eliminated with the faeces as metacyclic trypomastigotes and once inside the host, infect and penetrate cells and differentiate into intracellular replicative amastigotes until the cell is completely swollen and then transform back to trypomastigotes, lyse the cell and release them into the bloodstream completing the cycle^4–6^. These adaptations have been determined mainly as morphological changes but very little is known about the molecular and metabolic processes necessary through the life cycle to produce these changes^7^.

Contrary to most eukaryotes, *T. cruzi*, as other kinetoplastids, does not regulate their gene expression by the differential recruitment of polymerase II and just a few potential transcription factors have been described^8–11^. Instead, they transcribe long RNAs containing up to hundreds of genes also called polycistronic transcription units (PTUs)^12,13^. A second step of maturation consisting in the addition of a capped 39-nt spliced leader (SL) in the 5′ start and polyadenylation on the 3′ end, produces the individual and mature mRNAs^14^. In other species, such as *Leishmania*, has been demonstrated that transcription initiation preferentially occurs at divergent strand-switch regions (SSR), where PTUs raise in opposite directions on opposing DNA strands^15,16^.

While other kinetoplastids that cause human diseases such as *Leishmania* (Leishmaniasis) and *T. brucei* (sleeping sickness) have been and are still extensively studied regarding genomics and transcriptomics, allowing the description of most of its molecular regulatory mechanisms through its life cycle and host interaction, *T. cruzi* remains poorly understood^7^.

To date, some attempts to describe the global genome of *T. cruzi* have been performed but his high intra-species variability has made of this a complex challenge^17–21^. In recent years *T. cruzi* has gained some attention at transcriptomic and proteomic level^3,22–25^ but contrary to other kinetoplastids, little is known about the parasite molecular switches. In this work, we performed RNAseq analysis of Sylvio X10 strain to describe the trypomastigote transcriptome, but importantly it helped to improve significantly the previous genome sequence of this strain and also allow describing for the first time some of its main regulatory molecular mechanisms of *T. cruzi* mRNA processing.

## Results and Discussion

### Genome correction, transcriptome assembly and quantification

RNA from trypomastigotes of *T. cruzi* (strain Sylvio X10) was sequenced after polyA+ selection on Illumina MiSeq sequencing platform, generating 17,332,912 of paired-end reads (length: 75 nucleotides). Raw data was deposited to the SRA database under accession number **PRJNA546488**. Raw reads trimmed and filtered (maximum and minimum length: 100 and 50 respectively, minimum mean quality 25, phred score based) were mapped to SylvioX10 genome deposited on Tritryp (http://tritrypdb.org/, “TcruziSylvioX10-1”) allowing up to three mismatches with bowtie2. Considering that about 81.75% of the total reads were successfully mapped to the reference genome, we use it as *bona fide* genome reference.

The Sylvio X10 genome is composed by 47 “chromosome-like sequences” (C-LS) that are long scaffolds, no complete chromosomes. Thus, it is important to notice that non-aligned reads may correspond to undefined genomic regions (gaps) or missing chromosomes. Consequently, RNAseq reads were used to improve genomic assembly, such as the correction of deletions, insertions, SNPs etc. For this, we performed an assembly correction using Pilon, a bioinformatic tool for correcting drafted haploid and diploid microbial assemblies using paired end reads. Also, a previous paired DNAseq reads under the NCBI bioproject number PRJNA395140 were included to this genomic correction.

**Figure 1:**
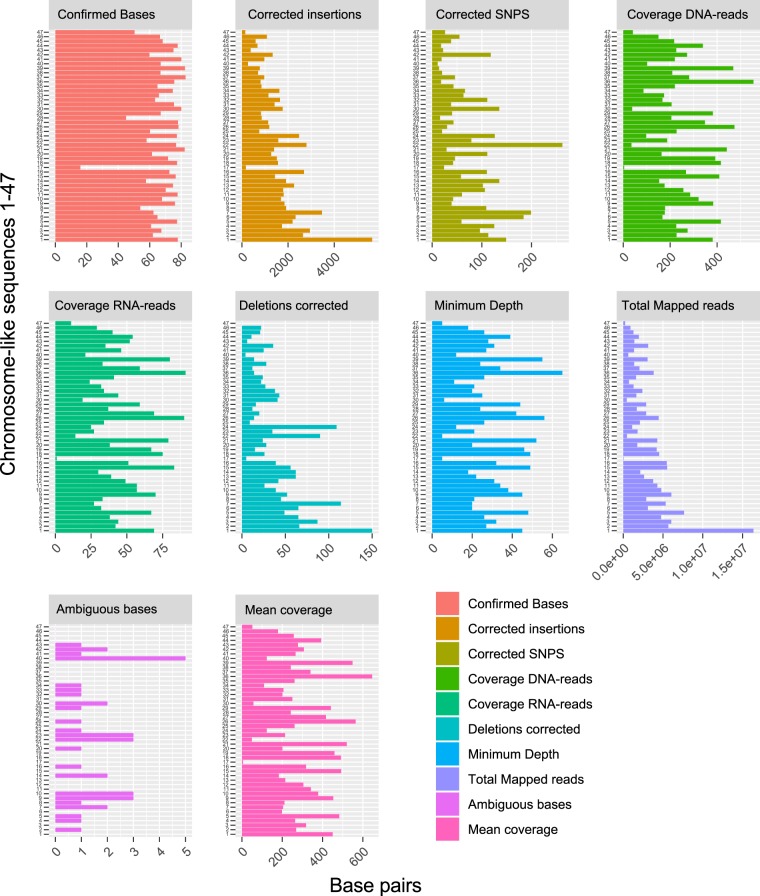
Genome improvement summary. Read depth mapping, coverage, and changes applied to the previous genomic sequence of Sylvio X10.

Results of Pilon improvement are summarized in Fig. 1. About 68% of the total genome bases were confirmed, corresponding to about 28 Mb of information (including gaps). Sequence correction affected mainly coding regions, 3,415 SNPs and 39 ambiguous bases were corrected, 72,887 insertions removed and 1,810 previous deletions were also corrected. Eighteen of the forty-seven C-LS decrease in total length up to 23 Kb as C-LS 4; the longest C-LS (1) decrease 7.2 Kb and the second longest C-LS decrease 12.8 Kb, while the shortest (C-LS 47) decreased 7.2 Kb. In total, 133.35 Kb of nucleotide insertions were trimmed from the previous assembly, while 43.66 Kb of new information was added to the new genome. In total, 177.02 Kb were corrected. In addition, it is important to notice that C-LS 17 and 47 had the lowest DNA-RNA sequencing coverage and the biggest ratio gaps per assembled base. This may be indicative that these sequences correspond to structural non-coding chromosomes, ambiguous assemblies or spurious sequences. But it clearly needs to be confirmed by further deeper genomic studies (Annotation in Supplementary File 1).

After genome correction 82.35% of the trimmed RNAseq reads were successfully mapped and were used in further analysis.

In trypanosomatids a discriminatory mechanism for the initiation of transcription at individual *loci* is absent in most genes. Thus, long RNAs containing more than one gene are transcribed at the same time by the polymerase II into long polycistronic transcription units (PTUs), requiring further mRNA maturation process that had been described before and named as trans-splicing and polyadenylation^26–28^. In this sense, the total transcripts were obtained in two steps: firstly, primary assembly (potential PTUs) was performed using Stringtie identifying 9,108 transcripts, corresponding to previous transcriptomic analysis^24,29^, but this is less than 50% of the recently predicted ORF’s (20,058) that constitutes the *T. cruzi* genome^21^, or even the total genes annotated in Sylvio X10 (20,619).

**Figure 2:**
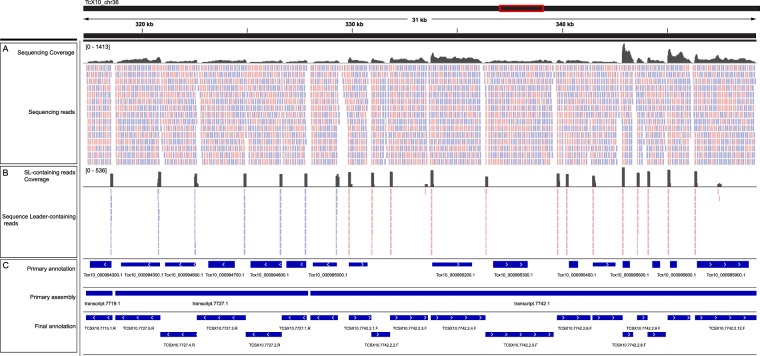
Transcriptomic assembly and annotation. Figure shows 31 kb spanning region of chromosome 2. (**A**) Sequencing coverage and reads aligned to reference genome, (**B**) Sequence Leader-containing reads mapped to reference, (**C**) Genomic and transcriptomics annotations: primary annotations corresponds to genomic annotations from companion and Stringtie, primary assembly (Stringtie) and final transcriptomic annotations.

We cannot discard the presence of unprocessed and stable PTUs in *T. cruzi* in our primary assembly as it has been describe in some instances in tryponosomatids^30^. However, since translation regulation relays mainly on mRNA stability, we found potential mature messengers from the same PTUs showing clear different levels of abundance (Fig. 2A), some of them match to more than one predicted gene. Surprisingly, most of PTUs did not match to any predicted genes.

In order to define the mature transcripts, we took advantage of the 39-nucleotide mini-exon sequence (SL) and polyA tail present in the 5′ and 3′-UTRs respectively of all mRNAs-^31–33^, as described by Rastrojo *et al*.^34^. 1,086,343 reads containing the SL sequence were trimmed and aligned back to the reference genome identifying 42,700 total SL-insertion sites (Fig. 2B), whereas 85,452 reads containing polyA-reads and 14,512 poly-Adenylation addition sites were identified. SL-containing sequences aligning in contrary orientation in any single PTU, confirming that in *T. cruzi* trans-splicing occurs only in the same orientation of the PTU similar to what has been described in *Leishmania major Friedlin* transcriptome^34^.

Considering that the previous annotated genome sequence was modified by our RNA analysis, the new Sylvio X10 genome was re-annotated using Companion and our transcriptomic evidence.

Using the SL and polyA insertion sites to define the 5′ start and 3′ end, respectively, most of the primary assembled PTUs were finally divided into 18,666 mature mRNAs. Of these, 1,172 were annotated as polycistronic, corresponding to sequences that contain one or more annotated ORFs, but that were not divided into mature transcripts by the presence of SL or poly-Adenylation-containing reads. Eight hundred seventy-two were annotated as truncated transcripts, corresponding to transcripts that do not cover its entire predicted ORF length (5′ or 3′ sequence). Although polycistronic and truncated mRNAs needs to be confirmed by future genomic and transcriptomic analysis, most cases correspond to low coverage transcripts. This may simply reflect that they are low-abundant transcripts at trypomastigote stage.

The remaining reads (about 3 million paired-end) that did not map to the reference genome or to kinetoplastid known sequences, were assembled *de novo* into potential transcripts using rnaSPAdes. Total potential assembled transcripts were filtered by coverage (RDC >= 10), length (>1 kb), sequence redundancy (≥90%) and contained ORFs (just contigs containing one ORF were considered). Finally, we obtained a total of 4,311 extra-transcripts (About 9.62 Mb). The abundance of these transcripts were also calculated using Stringtie and the sequences were added to the final transcriptome obtaining a total of 22,977 potential mRNAs. Those transcripts were named using the same nomenclature in other trypanosomatids: *Trypanosoma cruzi* “TC”, Strain; “SX10”, Polycistron Number, Monocistron number and Sense; “F”, “R” or “X” (no sense identified) e.g TCSX10.1.1.F. *De novo* assembled transcripts were named using the contig number instead of polycistron and monocistron numbers, by default sorted by length (increasing order) and adding “ND” (non-defined) e.g TCSX10_ND.1.F, the sense was defined by the encoded ORF.

**Figure 3:**
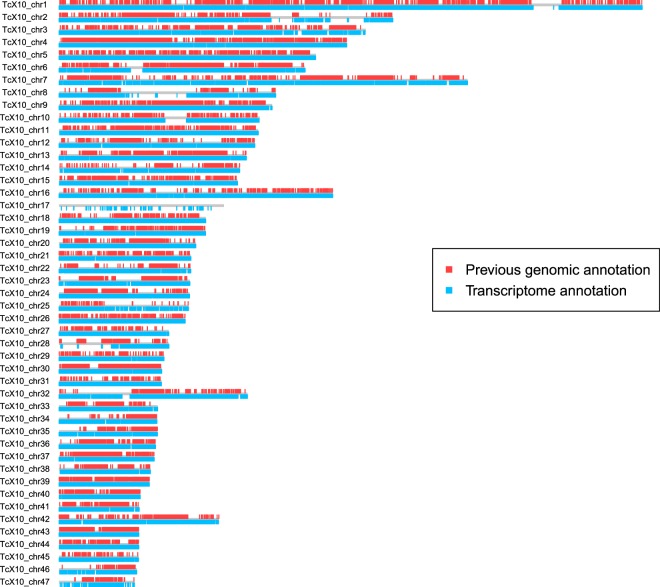
Coding sequence of Sylvio X10 C-LS. Previously defined (by genomic annotation) and the coding sequence defined by RNAseq data.

Taken together our results indicate that about the 79.95% of the *T. cruzi* genome (strain Sylvio X10) corresponds to coding sequence and therefore about 20% correspond to non-conding structural sequences. This is in sharply contrast with previous genomic analysis were only 37.73% of the total genome were defined as corresponding to coding sequences. Thus, our results have more than duplicated this estimation, largely improving the previous genomic annotation (Fig. 3). Besides, taking into account the length of ND transcripts, the haploid genome for Sylvio X10 may be higher than previously reported (at least 51 Mb).

In related species such as *Leishmania*, transcription initiation preferentially occurs at divergent strand-switch regions (SSR), where PTUs raise in opposite directions on opposing DNA strands^15,16^. In the Sylvio trypomastigote stage, we have identified a total of 226 SSR transcription initiation sites across the entire genome and up to 15 in a single C-LS (TcX10_chr24). All C-LSs contains at least one SSR (except TcX10_chr17) and 4.8 on average per chromosome. There is a medium positive correlation (0.54) between the number of SSR and C-LS length. On the other hand, there is a strong positive correlation between the total transcripts per chromosome and the truncated/polycistronic (0.86 and 0.84, respectively) transcripts found (as expected), whilst, there is no significant correlation between the number of forward, reverse and undefined transcripts (Fig. S1).

Seventeen thousand three hundred thirty-four transcripts have a FPKM (fragments per kilobase per million mapped reads) greater than or equal to 10 and quartile 3 includes values up to 66, whereas 59 transcripts have the maximum values (outliers, FPKM greater than or equal to 1,000) as shown in Fig. S2. These 59 top abundant transcripts (with FPKM greater or equal to 1,000) and its predicted function are listed in Table 1. Surprisingly, 83% (49) of the 59 more abundant transcripts correspond to *de novo* assembled transcripts and therefore were not annotated on the actual available genome for this strain.

**Table 1:** The top 59 abundant transcripts in SylvioX10 transcriptome and predicted functions.

Transcript Name	FPKM	Blastx-Hit	Accession Number
TCSX10_ND.1840.F	7377.97	Elongation factor 1-alpha (EF-1-alpha)	XP_819439.1
TCSX10_ND.7941.F	4776.96	Mucin-like glycoprotein	XP_803248.1
TCSX10_ND.3154.F	4669.15	D-isomer specific 2-hydroxyacid dehydrogenase-protein	XP_805652.1
TCSX10_ND.1768.F	4298.94	Heat shock protein 85	XP_814892.1
TCSX10_ND.5081.F	3981.67	Mucin-like glycoprotein	XP_805628.1
TCSX10_ND.4436.F	3773.12	Cystathionine beta-synthase	XP_820988.1
TCSX10_ND.6083.F	3120.83	Heat shock protein 70 (HSP70)	XP_817738.1
TCSX10_ND.4352.F	2740.59	Histone H2B	XP_805384.1
TCSX10.6560.6.R	2501.74	Hypothetical protein	XP_815965.1
TCSX10_ND.5435.F	2434.05	L-threonine 3-dehydrogenase	XP_812904.1
TCSX10_ND.5069.F	2373.31	Trans-sialidase	XP_820072.1
TCSX10_ND.7601.F	2301.47	RNA-binding protein	XP_819301.1
TCSX10_ND.2296.F	1981.37	Serine carboxypeptidase (CBP1)	XP_817769.1
TCSX10_ND.6353.F	1942.05	60S ribosomal protein L10a	XP_821884.1
TCSX10.8474.1.R	1921.65	Mucin-like glycoprotein	XP_804744.1
TCSX10_ND.466.F	1916.11	Amino acid transporter	XP_804156.1
TCSX10_ND.6832.F	1890.20	Glutamamyl carboxypeptidase	XP_804561.1
TCSX10_ND.364.F	1838.19	Hexose transporter	XP_814821.1
TCSX10_ND.3852.F	1784.70	Putative helicase	N/A
TCSX10.4107.1.R	1619.73	40S ribosomal protein S33	XP_810278.1
TCSX10_ND.3412.F	1616.62	Tyrosine aminotransferase	XP_821468.1
TCSX10_ND.5699.F	1611.10	Hypothetical protein	XP_808955.1
TCSX10_ND.3416.F	1605.96	Tyrosine aminotransferase	XP_821468.1
TCSX10.3691.1.F	1570.57	60S ribosomal protein L44	XP_804017.1
TCSX10_ND.7216.F	1551.15	Hypothetical protein	XP_814544.1
TCSX10_ND.1981.F	1549.12	40S ribosomal protein S21	XP_813246.1
TCSX10_ND.6160.F	1542.95	Sterol 24-c-methyltransferase	XP_802864.1
TCSX10_ND.7486.F	1431.42	Elongation factor 2	XP_809041.1
TCSX10_ND.5213.F	1429.50	Ubiquitin/ribosomal protein S27a	XP_817403.1
TCSX10_ND.7089.F	1428.85	60S acidic ribosomal protein P0	XP_821117.1
TCSX10_ND.6956.F	1373.65	60S ribosomal protein L5	XP_814693.1
TCSX10_ND.7682.F	1360.50	60S ribosomal protein L2	XP_816366.1
TCSX10_ND.326.F	1352.74	Hexose transporter	XP_814821.1
TCSX10_ND.8140.F	1352.57	Putative mucin TcSMUGS	N/A
TCSX10_ND.7552.F	1341.34	40S ribosomal protein S4	XP_815346.1
TCSX10_ND.3709.F	1316.95	Cysteine peptidase	XP_820174.1
TCSX10_ND.8348.F	1277.92	60S ribosomal protein L18	XP_819826.1
TCSX10_ND.2638.F	1255.85	Glycosomal phosphoenolpyruvate carboxykinase	XP_811627.1
TCSX10_ND.1562.F	1255.38	Glycosomal phosphoenolpyruvate carboxykinase	XP_811627.1
TCSX10.6382.2.F	1236.96	60S acidic ribosomal protein P2 beta (H6.4)	XP_806207.1
TCSX10_ND.6305.F	1221.18	Tryparedoxin peroxidase	XP_802803.1
TCSX10.5222.9.F	1216.11	Kinetoplastid membrane protein KMP-11	XP_810488.1
TCSX10_ND.8087.F	1215.93	Ribosomal protein S25	XP_819714.1
TCSX10_ND.5427.F	1163.64	Heat shock 70 kDa protein	XP_806221.1
TCSX10_ND.220.F	1162.89	P-type H+-ATPase	XP_010697981.1
TCSX10_ND.4125.F	1154.22	90 kDa surface protein	XP_814897.1
TCSX10_ND.5464.F	1145.67	Trans-sialidase	XP_804567.1
TCSX10_ND.4595.F	1143.34	Enolase	XP_819700.1
TCSX10_ND.4559.F	1130.09	Histone H2A	XP_819378.1
TCSX10.457.12.R	1127.90	Ribosomal proteins L37	XP_819516.1
TCSX10_ND.5709.F	1072.75	Hypothetical protein	XP_815965.1
TCSX10_ND.4984.F	1067.71	ADP,ATP carrier protein 1	XP_812264.1
TCSX10_ND.8347.F	1056.89	Trans-sialidase	XP_809719.1
TCSX10_ND.6727.F	1056.79	Heat shock 70 kDa protein	XP_804120.1
TCSX10.5300.1.R	1026.09	40S ribosomal protein S11	XP_809317.1
TCSX10.5088.14.F	1022.97	ribosomal protein L24	XP_820052.1
TCSX10_ND.8088.F	1009.79	40S ribosomal protein SA	XP_805747.1
TCSX10.1814.2.R	1006.31	60S ribosomal protein L17	XP_820080.1
TCSX10_ND.7034.F	1000.45	60S ribosomal protein L7a	XP_809485.1

The top abundant transcript corresponds to a ND transcript (TCSX10_ND.1840.F) containing an ORF coding for a highly conserved (in *T. cruzi*) elongation factor 1-alpha (EF-1-alpha). Different roles have been assigned to EF-1-alpha in various cellular processes, including metabolism, cytoskeletal organization, oncogenic transformation, apoptosis and anti-apoptosis and in *T. cruzi* has been described as potential regulator of gene expression^35^. Interestingly a second elongation factor (EF-2, TCSX10_ND.7486.F) was found in this top abundant list. The second and fifth top abundant transcripts correspond to mucin-like glycoproteins (TCSX10_ND.7941.F and and TCSX10_ND.5081.F) that are likely associated with parasite cell invasion and survival in Tc I strains^36^. Furthermore, 4 of these top abundant mRNAs in Sylvio X10 corresponds to heat shock proteins, as occurs in *Leishmania* (promastigotes) where they make up to 2.1% of the total protein in unstressed conditions^37^.

RNA binding proteins (RBPs), due their role in regulation of gene expression may represent one of the most important gene family for which parasite stage differential expression/transcript abundance information is of special interest. Unexpectedly, we found just one transcript (TCSX10_ND.7601.F, FPKM = 2301.46829) showing RNA recognition motif 3 (RRM3) of type I (polyA-binding proteins: PABPs) which is conserved in proteins that bind to the poly(A) tail of most eukaryotic mRNAs^38^. It may constitute a potential drug target for a translational inhibitor at trypomastigote stage that deserve deeper analysis across the entire parasite’s life cycle. Additionally, we found being part of this list 17 mRNAs coding for ribosomal proteins (9 for 60S, 5 for 40S and one for S25, L24 and L37). It has been demonstrated that striking differences in ribosomal composition of trypanosomatids comparing to other eukaryotes (mainly the mammal host) may lead also to specific drug targets in Chagas disease^39–41^. The remaining abundant transcripts encode canonical histones H2B and H2A (TCSX10_ND.4352.F, TCSX10_ND.4559.F) and some others corresponding to proteins involved in cell metabolism.

The minimum transcript length was of 200 nucleotides and the maximum about 20.5 Kb (TCSX10.4085.2.F, a polycistronic transcript not divided by SL-containing or polyA-containing reads but containing 2 ORFs) and TCSX10_ND.1.F (a *de novo* assembled) transcript of about 20.2 Kb containing an ORF of 6,635 residues encoding a conserved transferase. The predominant length for Sylvio X10 transcripts is from 800 nucleotides to 2.2 Kb.

Finally, 12,146 of the C-LS transcripts (65%) and 4,197 (97%) of the ND transcripts were successfully identified by blastx hit to nr protein database, non-blasted transcripts were annotated as “Not Found”.

### Functional enrichment analysis

**Figure 4:**
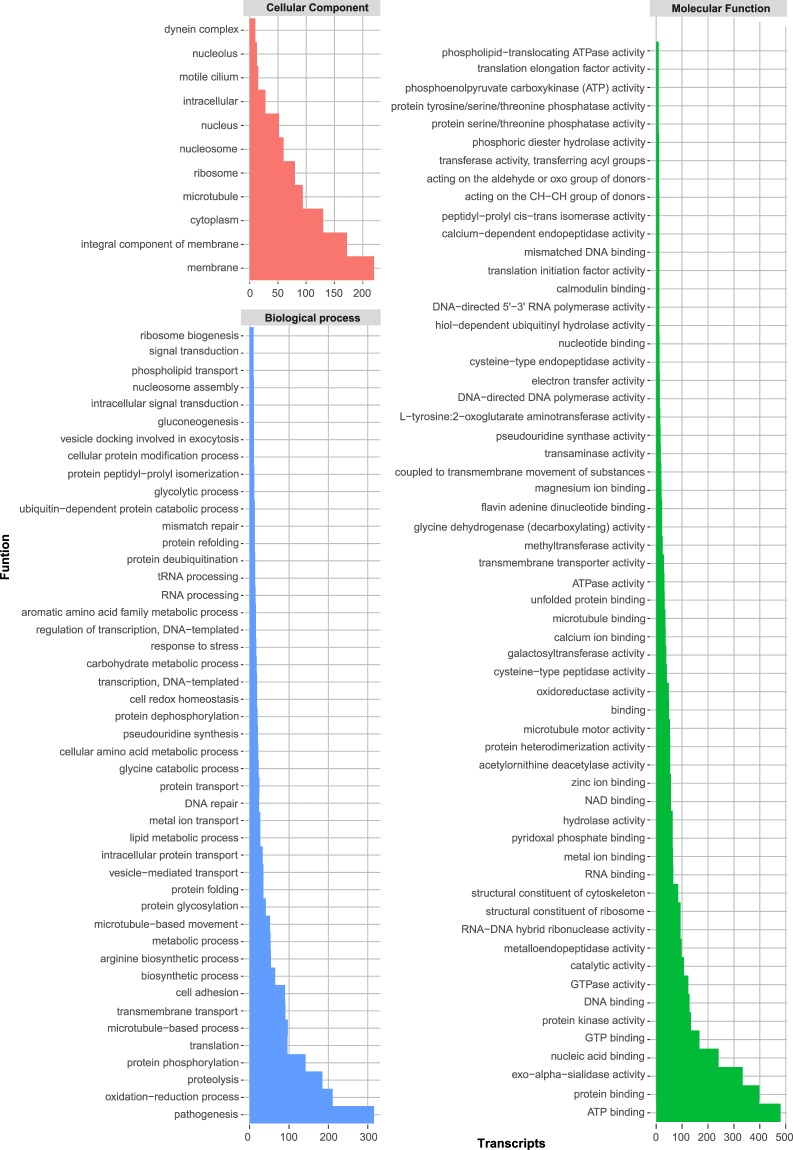
Gene ontology classification (Level three) of the Sylvio X10 transcriptome. Families containing at least 10 mapped transcripts were considered.

In order to define and visualize their functions, mature transcripts obtained in this study were ascribed gene ontology IDs (GO terms) with the Blast2GO tool. Level 3 of the gene ontology (GO terms) families mapped (at least 10 transcripts to be considered significant) were used to visualize the complete classification and functional enrichment as show in Fig. 4. In agreement to previous results, we found a set of transcripts ubiquitously abundant on epimastigotes, amastigotes and trypomastigotes^24^: 149 related to microtubule movement/processes (biological process/molecular function), 125 for chromosome organization/DNA packing (cellular component) and 17 for stress response (biological process) among others.

In Sylvio X10 trypomastigotes, the most abundant transcripts corresponding to cellular components belongs to membrane products (up to 200), cytoplasm and ribosomes. A total of 1,198 transcripts were classified into this level. GO terms associated to biological process corresponds to specific objectives that the organism is genetically programmed to achieve, including mRNAs that are implicated along the complete process or in the final outcome. A total 2,812 transcripts correspond to this group. Since the sequenced RNA was extracted from trypomastigotes (non-replicative infective form), is not surprising that the most represented biological process corresponds to pathogenesis (316), proteolysis (185), protein phosphorylation (142), cell adhesion (90) necessary in infection steps. These findings are also in agreement to Berná *et al*.^24^, which describes the enrichment of processes related to movement, adhesion, invasion and signalling in trypomastigotes (undefined strain). GO terms of Molecular function include transcripts involved on specific activities, and we found that in the Sylvio X10 transcriptome this is the GO family that includes most of the transcripts mapped (4,624). ATP-binding (480), protein-binding also known as glycoprotein-binding (398), exo-alpha-sialidase activity (334), protein kinases (134) and nucleic acid binding constitutes the top 5 most enriched functions.

Additionally, to the transcriptomic classification by GO terms, a second functional-based classification allow us the identification of the most abundant gene families over-represented across the entire transcriptome (Fig. S3). A total of 1,632 functionally related gene families were identified (17,929 transcripts), comprising 239 families (containing 10 or more genes) and corresponding to about the 52% (11,960) of the total transcriptome. Interestingly, 928 transcripts matching to Interpro GO ids did not show similar functions to other transcripts (single family) and 5,048 were not identified. This grouping of transcripts into functional families confirms that the trypomastigote physiological needs are covered by the transcription of multi-copy genes. As expected, some of these families correspond to the most abundant genes annotated along the *T. cruzi* genome^21^ such as; 1,329 transcripts coding for sialidase activity, 235 mucin-like proteins, 284 RHS (retrotransposon hot-spot) and 338 kinase proteins. Interestingly, about 428 transcripts corresponded to WD40-repeat-containing domain superfamily, proteins which are found in all eukaryotes and implicated in a variety of functions ranging from signal transduction and transcription regulation to cell cycle and apoptosis^42^. Specifically, WD40 motifs may act as a site for protein-protein interaction, and proteins containing WD40 repeats are known to serve as platforms for the assembly of protein complexes or mediators of transient interplay among other proteins. Other interesting abundant functionally related families are the P-loop containing nucleoside triphosphate hydrolase (695 transcripts), which is composed by proteins containing the most frequent domain of nucleotide-binding proteins^43^. This superfamily is characterized by the concanavalin A-like lectin/glucanase domain which includes proteins like glycosyl hydrolases, lectins, lyases, Beta-D-xylosidases, vp4 sialic acid binding protein among others^44^.

### Trans-splicing and poly-Adenylation sites

The addition of about 39-nt mini-exon (SL) to the 5′ start of all mRNAs is an essential part of the maturation process in trypanosomatids^45,46^. As a result, an AG dinucleotide has been described as the consensus sequence for SL trans-splicing, but unfortunately, no specific signal for polyadenylation had been defined in *Trypanosomatids*, just a global polypyrimidine tracts of variable length is supposed to compose these regions^34,47–50^. Small differences between *Leishmania* and *T. brucei* in nucleotide composition surrounding the AG nucleotides and polyadenylation sites have been described before, suggesting that slightly different specific mechanisms may control the mRNA maturation process across trypanosomatid species^34,48^. In contrast, in-depth analysis of these sites in *T. cruzi* has not been performed to date. Thus, in this work, analysis of SL-containing and polyA-containing reads allowed us to differentiate between mature transcripts and long RNAs or PTUs, but also to analyse trans-splicing and polyadenylation sites to single nucleotide resolution.

**Figure 5:**
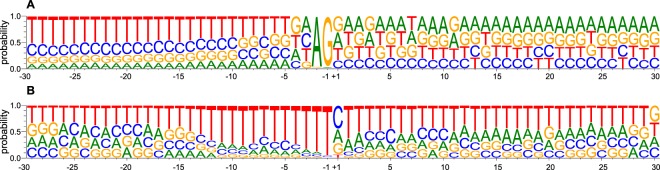
Genomic composition (by nucleotide frequencies) on SL and polyadenylation insertion sites. (**A**) SL addition sites (n = 18,589). (**B**) PolyA-tail addition sites (n = 9,789).

We search for sequence enrichment/motifs associated to SL-addition sites by the calculation of the weblogo sequence on the ±30 nucleotide region from the trans-splicing and polyadenylation sites (Fig. 5). A total of 18,589 SL-addition sites were found. In agreement to previous results^34,48^, the AG dinucleotide was determined as the conserved insertion signature with 97.39% and 97.19% of probability for each base, respectively (Fig. 5A). Surprisingly, in contrast to *Leishmania*^34^ (having a Cytosine) but concordantly with *T. brucei*^48^, an Adenine nucleotide was the most probable (31.84%) residue before the dinucleotide AG (position -3), followed by Cytosine (29.18%), Thymine (26.53%) and Guanine (12.42%), interestingly at position -4 Guanine is the most probable nucleotide (31.20%) as occurs in *L. major* but contrary to *T. brucei* where a polyT tract starts and continue as the most frequent composition up to 50 nucleotides upstream.

The most marked differences between *T. cruzi* with *L. major* and *T. brucei* in the DNA regions surrounding the SL addition sites are in the pyrimidine nucleotide enrichment. Whereas in *Leishmania* C-T dinucleotides clearly consist about the 70% of nucleotides present upstream and downstream the AG dinucleotide, in *T. cruzi*, this pattern is different. *T. cruzi* just conserved the C-T pattern in the upstream 5′ region while downstream region is composed of purine nucleotides (A-G) up to 60% (Fig. 5A). *T. brucei*, also only had the C-T pattern in the upstream 5′ region but downstream A-T dinucleotides are the most frequent bases, indicating that in general, *T. cruzi* transcripts show more proportional nucleotide composition and G + C profiles (as ocurr in *L. major*) than *T. brucei*.

Regarding the polyadenylation signals, trypanosomatids, contrary to other eukaryotes, do not share the conserved AAUAA polyadenylation motif^51,52^. In contrast, an A-T, A-T, A-T in *T. bruce*i and A-G, A-T, A-T for *L. major* have been described as the most probable dinucleotides preceding the polyadenylation site. In addition, sequence heterogeneity surrounding ±40 bases of these motifs between these *T. cruzi* related species have been determined. *L. major* displays a more variable sequence composition upstream and downstream polyadenylation site (specially the 5′)^34,47^ whilst, in *T. brucei* is possible to visualize a uniform pattern in both extremes composed by T-A nucleotides. In the *T. cruzi*, we found and analysed 9,789 polyadenylation sites that allow us to identify and visualize the surrounding genomic region (Fig. 5B).

Interestingly and contrary to other trypanosomatids where a AA dinucleotide has been found on the polyadenylation site^34,53^, we found that a single nucleotide is the most probable signal of polyadenylation start. Cytosine is the most frequent signalling nucleotide (45.32%), Adenine and Guanine are almost equally frequent bases (26.34% and 21.52%, respectively) and Thymine is the less frequent nucleotide with 6.79%. Likewise, our analysis allowed us to identify abundant Thymine composition (up to 95.45% at position −1) in upstream region, and a higher T + A composition (up to 89%) on the downstream sites, starting from +2 position. Together, our results indicate that mRNA maturation processes in *T. cruzi* may differ significantly from its closely related kinetoplastid counterparts.

## Conclusions

In this work and for first time in *T. cruzi*, we have described some of the most important transcriptomic features in the tripomastigote stage of Sylvio X10 strain, as well as to contribute to the improvement of its genome sequence. Firstly, we corrected about 177 Kb of ambiguous genomic regions using RNAseq data and secondly we identified, classified, quantified and annotated 22,977 mature mRNAs. Their identification and quantification has allowed us to identify the main probable regulatory elements, and to significantly improve the entire genomic annotation.

We have also identified the transcription initiation SSRs across the genome, and determined that are positively correlated to the “chromosomal” length and therefore there is no strand-specificity as it occurs in *Leishmania*. Slighter abundance of Forward transcripts, also suggest that PTUs in *T. cruzi* are equally transcribed and that according to the genomic annotation the packaged genes may be or not functionally related. Besides, our results have identified that Sylvio X10 trypomastigotes exhibit a predominance of surface protein mRNAS, mainly those encoding trans-sialidase and mucin-like proteins which also constitutes the most expanded gene families, confirming that the gene copy number act as secondary regulator mechanism of protein expression.

Finally, but equally important, we have identified and described the trans-splicing and polyadenylation sites in *T. cruzi* which are different to other closely related kinetoplastids such as *Leishmania* and *T. brucei*, mainly on the polyadenylation signal and surrounding sequences.

## Material and Methods

### Parasite cultures and RNA extraction

The Sylvio X10 strain was obtained from Dr. M. Miles (London School of Hygiene and Tropical Medicine, London, UK) through the European program ChagasEpiNet. Vero cells were grown in RPMI medium supplemented with 5% fetal bovine serum (FBS), 100 UI/mL of antibiotics mixture, 10 µg/mL streptomycin and 2 mM glutamine at 37 °C in an atmosphere of 5% CO2 until the cells reached 80% confluence. The cell monolayer was subsequently infected with metacyclic trypomastigotes of *T. cruzi* Sylvio X10 strain. After 4 days, the supernatant medium was collected, Vero cells and amastigotes were removed by centrifugation at 1000 g by 5 minutes. Trypomastigotes were collected by centrifugation at 1600 g for 10 minutes. Three biological replicates where mixed before RNA isolation.

RNA was isolated using the RNeasy Mini Kit (Qiagen) and treated with RNAse-free DNAse I. RNA samples were quantified by absorbance at 260 nm using the Nanodrop ND-1000 (Thermo Scientific), all samples showed an A260/A280 ratio higher than 2.0. In addition, RNA integrity was checked in a bioanalyzer (Agilent 2100) resulting on RIN value higher than 8.

### RNAseq and transcriptome analysis

RNA-seq was performed at the Massive Sequencing Platform of Cantoblanco (CSIC-PCM, Madrid, Spain). Standard libraries for massive sequencing were generated using the TruSeq RNA Sample Prep Kit (Illumina). Briefly, poly-A + RNA was selected by oligo-dT chromatography, and RNA fragmentation was achieved using divalent cations under elevated temperature. Afterwards, these fragments were used to generate a cDNA library, and cDNA fragments corresponding in size to about 450–550 bp were isolated from an agarose gel. Paired-end reads of 100 nucleotides were obtained, and raw reads were subject to quality-filtered using the standard Illumina process and analysed using FASTQC tool^54^.

Reads were mapped to the reference genome using Bowtie2^55^, transcript assembly and abundance was calculated with Stringtie^56^ and assembly corrections were performed using Pilon^57^. Transcript identification/classification was performed by Blas2GO suite^58^, Blastx using the protozoan non-redundant (nr) proteome downloaded from the NCBI and in-house python scripts. Trans-splicing and polyadenylation sites were identified as described before by Rastrojo *et al*.^34^ and the local version of the WebLogo tool^59^. All figures and statistical analysis were performed using R and Rstudio^60^.

Additional bioinformatics tools were used to handle, parse, analyse or visualize sequencing data such as samtools^61^, CD-HIT^62^ and IGV^63^.

## Supplementary information

Supplementary Figures

Supplementary file 1

## Data Availability

Raw data has been submitted to the NCBI repository (SRA) under the accession number PRJNA546488. The new genome version, transcript sequences and annotation file have been also submitted to the TritrypDB for its public access. Both data will be immediately available after the publication of our manuscript.

## References

[1] Bern C (2007). Evaluation and Treatment of Chagas Disease in the United States. JAMA.

[2] Chagas C (1909). Nova tripanozomiaze humana. Estudos sobre a morfolojia e o ciclo evolutivo de Schizotrypanum cruzi n. gen., n. sp., ajente etiolojico de nova entidade morbida do homen. Mem Inst Oswaldo Cruz.

[3] Li Y (2016). Transcriptome Remodeling in Trypanosoma cruzi and Human Cells during Intracellular Infection. PLOS Pathog..

[4] Tibayrenc, M. & Telleria, J. *American trypanosomiasis: Chagas disease: one hundred years of research*. (Elsevier, 2010).

[5] Rassi A, Rassi A, Marcondes de Rezende J (2012). American Trypanosomiasis (Chagas Disease). Infect. Dis. Clin. North Am..

[6] Echeverria LE, Morillo CA (2019). American Trypanosomiasis (Chagas Disease). Infect. Dis. Clin. North Am..

[7] Patino LH, Ramírez JD (2017). RNA-seq in kinetoplastids: A powerful tool for the understanding of the biology and host-pathogen interactions. Infect. Genet. Evol..

[8] Sabalette KB (2019). The RNA-binding protein TcUBP1 up-regulates an RNA regulon for a cell surface-associated Trypanosoma cruzi glycoprotein and promotes parasite infectivity Downloaded from. J. Biol. Chem.

[9] Srivastava A, Badjatia N, Lee JH, Hao B, Günzl A (2018). An RNA polymerase II-associated TFIIF-like complex is indispensable for SL RNA gene transcription in Trypanosoma brucei. Nucleic Acids Res..

[10] Weisbarth RT (2018). The Trypanosoma cruzi RNA-binding protein RBP42 is expressed in the cytoplasm throughout the life cycle of the parasite. Parasitol. Res..

[11] Das A (2017). An essential domain of an early-diverged RNA polymerase II functions to accurately decode a primitive chromatin landscape. Nucleic Acids Res..

[12] El-Sayed NM (2004). Comparative Genomics of Trypanosomatid Parasitic Protozoa. Science (80-.)..

[13] De Gaudenzi JG, Noe G, Campo VA, Frasch AC, Cassola A (2011). Gene expression regulation in trypanosomatids. Essays Biochem.

[14] Sutton RE, Boothroyd JC (1986). Evidence for Trans Splicing in Trypanosomes. Cell.

[15] Martínez-Calvillo S, Nguyen D, Stuart K, Myler PJ (2004). Transcription Initiation and Termination on Leishmania major Chromosome 3. Eukaryot. Cell.

[16] Martínez-Calvillo, S. *et al*. Transcription of Leishmania major Friedlin Chromosome 1 Initiates in Both Directions within a Single Region. *Mol. Cell* **11** (2003). 10.1016/s1097-2765(03)00143-6 12769852

[17] Franzén O (2011). Shotgun sequencing analysis of Trypanosoma cruzi i Sylvio X10/1 and comparison with T. cruzi VI CL Brener. PLoS Negl. Trop. Dis..

[18] Najib M (2005). The Genome Sequence of Trypanosoma cruzi, Etiologic Agent of Chagas Disease. Science (80-.)..

[19] Reis-Cunha, J. L. *et al*. Chromosomal copy number variation reveals differential levels of genomic plasticity in distinct Trypanosoma cruzi strains. *BMC Genomics* **16** (2015). 10.1186/s12864-015-1680-4 PMC4491234 26141959

[20] Bern, L. *et al*. Expanding an expanded genome: long-read sequencing of Trypanosoma cruzi, 10.1099/mgen.0.000177 (2019). 10.1099/mgen.0.000177 PMC5994713 29708484

[21] Callejas-Hernández F, Rastrojo A, Poveda C, Gironès N, Fresno M (2018). Genomic assemblies of newly sequenced Trypanosoma cruzi strains reveal new genomic expansion and greater complexity. Sci. Rep..

[22] Chávez Santiago, Eastman Guillermo, Smircich Pablo, Becco Lorena Lourdes, Oliveira-Rizzo Carolina, Fort Rafael, Potenza Mariana, Garat Beatriz, Sotelo-Silveira José Roberto, Duhagon María Ana (2017). Transcriptome-wide analysis of the Trypanosoma cruzi proliferative cycle identifies the periodically expressed mRNAs and their multiple levels of control. PLOS ONE.

[23] Mara Bezerra dos Santos C (2018). Trypanosoma cruzi transcriptome during axenic epimastigote growth curve. Mem Inst Oswaldo Cruz, Rio Janeiro.

[24] Robello, C. *et al*. Transcriptomic analysis reveals metabolic switches and surface remodeling as key processes for stage transition in Trypanosoma cruzi. *PeerJ* **5** (2017). 10.7717/peerj.3017 PMC5345387 28286708

[25] Herreros-Cabello Alfonso, Callejas-Hernández Francisco, Fresno Manuel, Gironès Núria (2019). Comparative proteomic analysis of trypomastigotes from Trypanosoma cruzi strains with different pathogenicity. Infection, Genetics and Evolution.

[26] Queiroz Rafael, Benz Corinna, Fellenberg Kurt, Hoheisel Jörg D, Clayton Christine (2009). Transcriptome analysis of differentiating trypanosomes reveals the existence of multiple post-transcriptional regulons. BMC Genomics.

[27] Kelly S., Kramer S., Schwede A., Maini P. K., Gull K., Carrington M. (2012). Genome organization is a major component of gene expression control in response to stress and during the cell division cycle in trypanosomes. Open Biology.

[28] Clayton Christine (2013). The Regulation of Trypanosome Gene Expression by RNA-Binding Proteins. PLoS Pathogens.

[29] Minning, T. A., Weatherly, D. B., Atwood Iii, J., Orlando, R. & Tarleton, R. L. The steady-state transcriptome of the four major life-cycle stages of Trypanosoma cruzi. *BMC Genomics* **10** (2009). 10.1186/1471-2164-10-370 PMC2907688 19664227

[30] Jäger AV, De Gaudenzi JG, Cassola A, Frasch AC (2000). mRNA maturation by two-step trans-splicing/polyadenylation processing in trypanosomes. Natl. Acad. Sci..

[31] Borst P (1986). Discontinuous transcription and antigenic variation in trypanosomes. Ann. Rev. Biochem.

[32] Mccarthy-Burke, C., Taylor, Z. A. & Buck, G. A. Characterization of the spliced leader genes and transcripts in Tryymoma cruzi* (Chagas’ disease; truns-splicing; mRNA processing; RNA secondary structure SL gene, SL-RNA; Y-branch structure). *Gene* **82** (1989). 10.1016/0378-1119(89)90043-7 2684773

[33] Boothroyld J, Cross GA (1982). Transcripts coding for variant surface glycoproteins of Trypanosoma brucei have a short, identical exon at their 5′ end. Gene.

[34] Rastrojo Alberto, Carrasco-Ramiro Fernando, Martín Diana, Crespillo Antonio, Reguera Rosa M, Aguado Begoña, Requena Jose M (2013). The transcriptome of Leishmania major in the axenic promastigote stage: transcript annotation and relative expression levels by RNA-seq. BMC Genomics.

[35] Ronalte Alves, L., Oliveira, C. & Goldenberg, S. Eukaryotic translation elongation factor-1 alpha is associated with a specific subset of mRNAs in Trypanosoma cruzi. *BMC Microbiol*. **15** (2015). 10.1186/s12866-015-0436-2 PMC4436862 25986694

[36] David Ramírez, J., Jiménez, P., Jaimes, J. & Poveda, C. A systematic review of the Trypanosoma cruzi genetic heterogeneity, host immune response and genetic factors as plausible drivers of chronic chagasic cardiomyopathy. *Parasitology* **146** (2018). 10.1017/S0031182018001506 30210012

[37] Brandau S, Dresel A, Clos J (1995). High constitutive levels of heat-shock proteins in human-pathogenic parasites of the genus Leishmania. Biochem. J.

[38] Patel GP, Ma S, Bag J (2005). The autoregulatory translational control element of poly(A)-binding protein mRNA forms a heteromeric ribonucleoprotein complex. Nucleic Acids Res..

[39] Kamina AD, Williams N (2017). Ribosome Assembly in Trypanosomatids: A Novel Therapeutic Target. Trends Parasitol..

[40] Liu Z (2016). Structure and assembly model for the Trypanosoma cruzi 60S ribosomal subunit. PNAS.

[41] Brito Querido J (2017). The cryo-EM Structure of a Novel 40S Kinetoplastid-Specific Ribosomal Protein. Structure.

[42] Li D, Roberts R (2001). WD-repeat proteins: structure characteristics, biological function, and their involvement in human diseases. Cell. Mol. Life Sci..

[43] Leipe DD, Wolf YI, Koonin EV, Aravind L (2002). Classification and evolution of P-loop GTPases and related ATPases. J. Mol. Biol..

[44] Concanavalin A-like lectin/glucanase domain superfamily (IPR013320) &lt; InterPro &lt; EMBL-EBI. Available at, http://www.ebi.ac.uk/interpro/entry/IPR013320, (Accessed: 18th March 2019).

[45] Günzl A (2010). The Pre-mRNA Splicing Machinery of Trypanosomes: Complex or Simplified?. Eukaryot. Cell.

[46] MAIR GUNNAR, SHI HUAFANG, LI HONGJIE, DJIKENG APPOLINAIRE, AVILES HERNAN O., BISHOP JOSEPH R., FALCONE FRANCO H., GAVRILESCU CRISTINA, MONTGOMERY JACQUI L., SANTORI M. ISABEL, STERN LEAH S., WANG ZEFENG, ULLU ELISABETTA, TSCHUDI CHRISTIAN (2000). A new twist in trypanosome RNA metabolism: cis-splicing of pre-mRNA. RNA.

[47] Dillon LAL (2015). Transcriptomic profiling of gene expression and RNA processing during Leishmania major differentiation. Nucleic Acids Res..

[48] Kolev NG (2010). The transcriptome of the human pathogen Trypanosoma brucei at single-nucleotide resolution. PLoS Pathog..

[49] Xu, P., Wen, L., Benegal, G., Wang, X. & Buck, G. A. Identification of a spliced leader RNA binding protein from Trypanosoma cruzi. *Molecular & Biochemical Parasitology* **112** (2001). 10.1016/s0166-6851(00)00341-8 11166385

[50] Huang J, Van Der Ploeg LHT (1991). Requirement of a polypyrimidine tract for trans-splicing in trypanosomes: discriminating the PARP promoter from the immediately adjacent 3′ splice acceptor site. EMBO J..

[51] Beaudoing E, Freier S, Wyatt JR, Claverie J-M, Gautheret D (2000). Patterns of Variant Polyadenylation Signal Usage in Human Genes. Genome Res..

[52] Tian B, Hu J, Zhang H, Lutz CS (2005). A large-scale analysis of mRNA polyadenylation of human and mouse genes. Nucleic Acids Res..

[53] Wahle E, Keller W (1992). The biochemestry of 3′-end cleavage and polyadenylation of messengers rna precursors. Annu. Rev. Biochem..

[54] Babraham Bioinformatics - FastQC A Quality Control tool for High Throughput Sequence Data. Available at, http://www.bioinformatics.bbsrc.ac.uk/projects/fastqc/, (Accessed: 6th March 2017).

[55] Langmead, B. & Salzberg, L S. Fast gapped-read alignment with Bowtie 2. *Nat. Methods* **9** (2012). 10.1038/nmeth.1923 PMC3322381 22388286

[56] Pertea Mihaela, Pertea Geo M, Antonescu Corina M, Chang Tsung-Cheng, Mendell Joshua T, Salzberg Steven L (2015). StringTie enables improved reconstruction of a transcriptome from RNA-seq reads. Nature Biotechnology.

[57] Walker BJ, Abeel T, Shea T, Priest M, Abouelliel A (2014). Pilon: An Integrated Tool for Comprehensive Microbial Variant Detection and Genome Assembly Improvement. PLoS One.

[58] Gö Tz S (2008). High-throughput functional annotation and data mining with the Blast2GO suite. Nucleic Acids Res..

[59] Crooks, G. E., Hon, G., Chandonia, J.-M. & Brenner, S. E. WebLogo: A Sequence Logo Generator. *Genome Res*. **14** (2004). 10.1101/gr.849004 PMC419797 15173120

[60] RStudio Team (2015). RStudio Team (2015) RStudio: Integrated Development for R. RStudio, Inc. (2015). Available at, https://www.rstudio.com/, (Accessed: 21st March 2019).

[61] Li H (2009). The Sequence Alignment/Map format and SAMtools. Bioinforma. Appl. NOTE.

[62] Fu, L., Niu, B., Zhu, Z., Wu, S. & Li, W. Sequence analysis CD-HIT: accelerated for clustering the next-generation sequencing data. **28**, 3150–3152 (2012). 10.1093/bioinformatics/bts565 PMC3516142 23060610

[63] Robinson, J. T. *et al*. Integrative genomics viewer. *Nat. Biotechnol*. **29** (2011). 10.1038/nbt.1754 PMC3346182 21221095

