# Supplementary material for: Analysis of mRNA processing at whole transcriptome level, transcriptomic profile and genome sequence refinement of Trypanosoma cruzi
Source: Sci Rep. 2019 Nov 22;9:17376. doi: 10.1038/s41598-019-53924-6 (PMC6874640; doi:10.1038/s41598-019-53924-6)
Supplement: Supplementary file 1 — Supplementary Figures [file 41598_2019_53924_MOESM1_ESM.pdf]

Analysis of mRNA processing at whole transcriptome level, transcriptomic profile and genome sequence refinement of *Trypanosoma cruzi*.

Francisco Callejas-Hernández[1], Ángel Gutierrez-Nogues[1], Alberto Rastrojo[1], Núria Gironès*[1,2] and Manuel Fresno*[1,2]

1 Centro de Biología Molecular Severo Ochoa, Consejo Superior de Investigaciones Científicas, Universidad Autónoma de Madrid, Cantoblanco, Madrid, Spain.

2 Instituto Sanitario de Investigación Princesa, Madrid, Spain.

*Address correspondence to: mfresno@cbm.csic.es or ngirones@cbm.csic.es Tel: +34 91 1964565; Fax: +34 91 1964420;

Supplementary Information

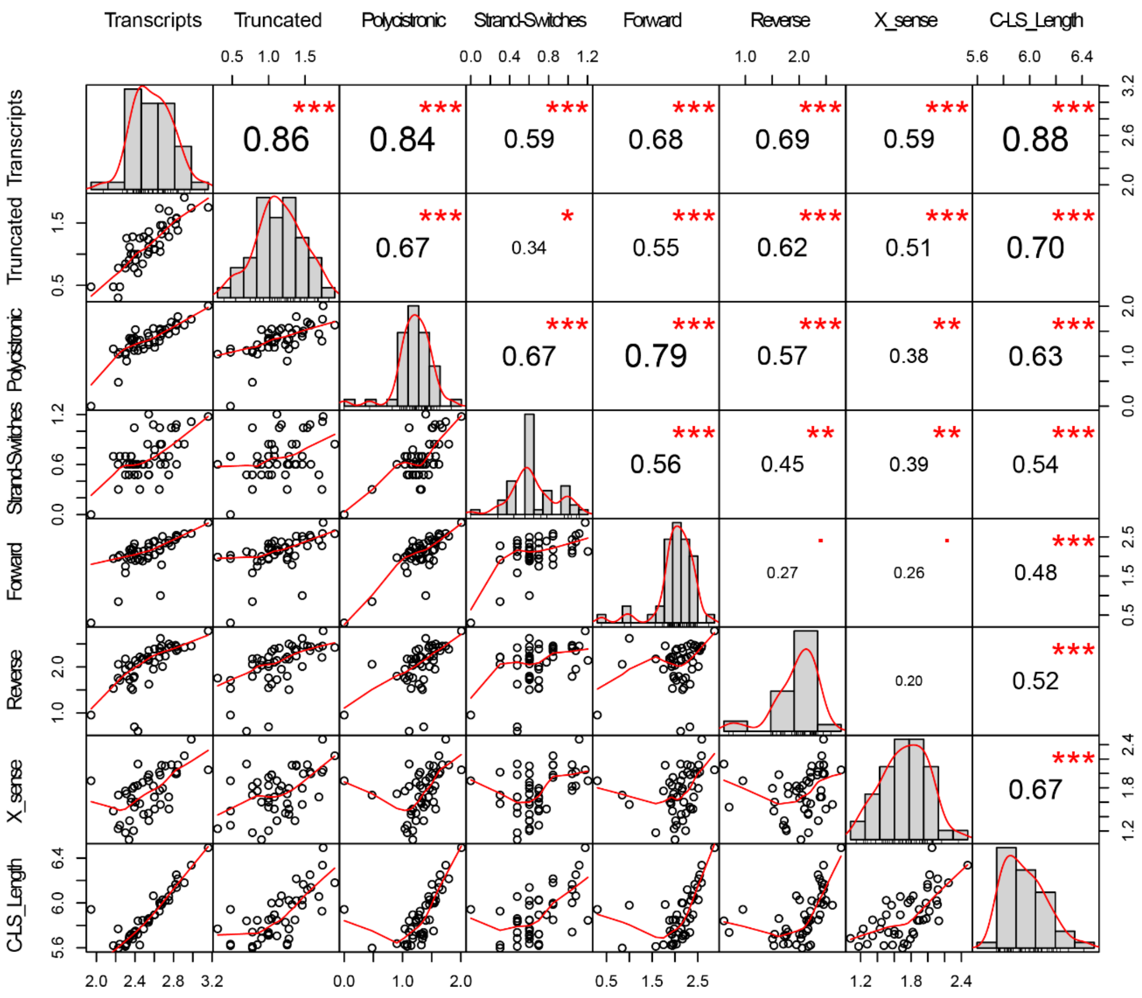

Supplementary Figure 1. Spearman correlation of Sylvio X10 transcriptome annotations. Spearman correlation between the annotated features of Sylvio X10

transcriptome, “transcripts”, correspond to total transcripts, the number of polycistronic and truncated transcripts per chromosome are labelled as “polycistronic” and “truncated”, respectively, “Forward”, “Reverse” and “X_sense” refers to the transcript sense, “Strand-switches” to the SSR identified and finally “C-LS_length” corresponds to the chromosomic length. Data distribution is shown in diagonal histograms and density lines, lower matrix contains the bivariate scatter plots with a fitted line and the correlation value is shown in the upper matrix. P-values of the correlation are shown as red stars (≤ to 0, 0.001, 0.01 corresponds to “***”, “**”, “*”, respectively).

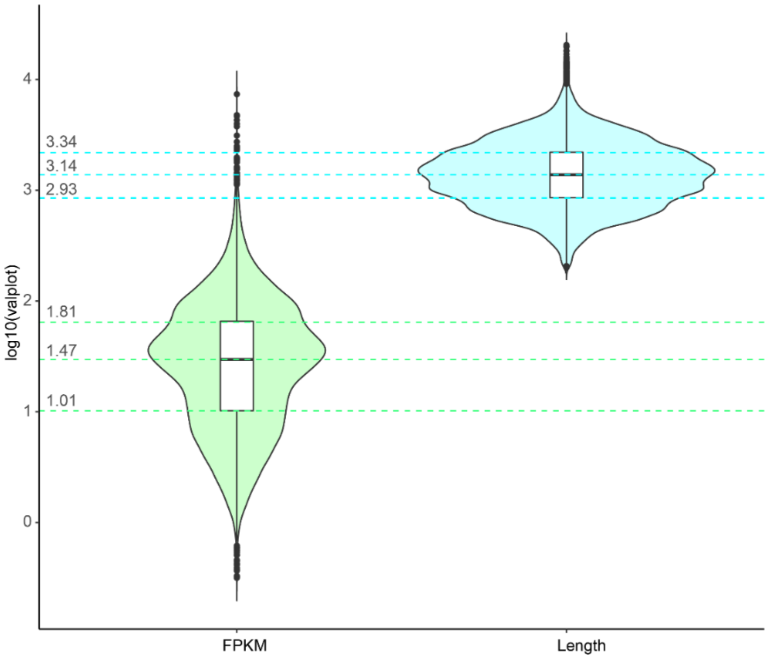

Supplementary Figure 2. FPKM values and length distribution of the complete transcriptome. Quartiles Q1, mean and Q3 are represented by dashed lines.

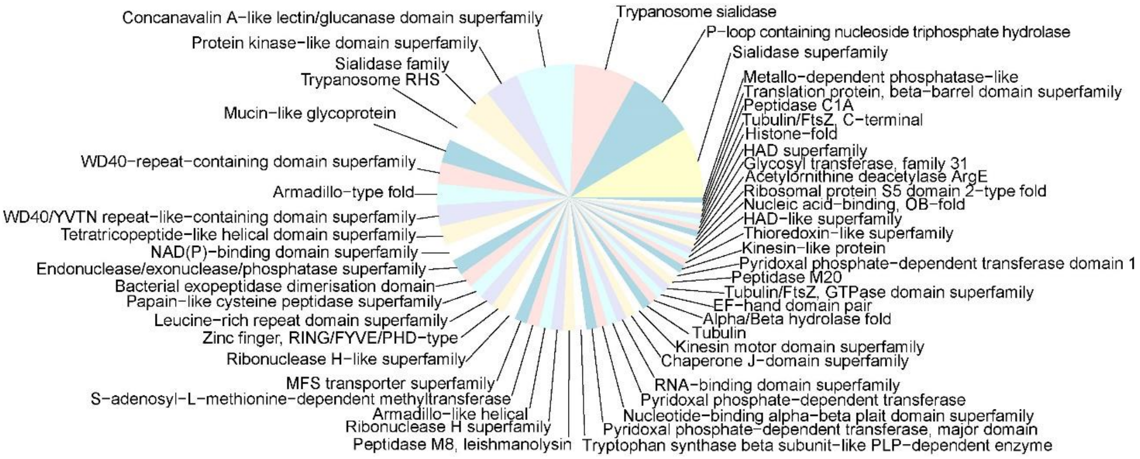

Supplementary Figure 3. More abundant transcripts in Sylvio X10 transcriptome. Multicopy gene families more abundant copy-number based in *T. cruzi* tripomastigotes.
